# A multimodality intervention to improve musculoskeletal health, function, metabolism, and well-being in spinal cord injury: study protocol for the FIT-SCI randomized controlled trial

**DOI:** 10.1186/s12891-022-05441-3

**Published:** 2022-05-25

**Authors:** K. F. Reid, T. W. Storer, K. M. Pencina, R. Valderrabano, N. K. Latham, L. Wilson, C. Ghattas, R. Dixon, A. Nunes, N. Bajdek, G. Huang, S. E. Skeels, A. P. Lin, S. M. Merugumala, H. J. Liao, M. L. Bouxsein, R. D. Zafonte, S. Bhasin

**Affiliations:** 1 Research Program in Men’s Health: Aging and Metabolism, Boston Claude D. Pepper Older Americans Independence Center for Function Promoting Therapies, Brigham and Women’s Hospital, Harvard Medical School, Boston, MA USA; 2 Center for Clinical Spectroscopy, Department of Radiology, Brigham and Women’s Hospital, Harvard Medical School, Boston, MA USA; 3 Center for Advanced Orthopaedic Studies, Beth Israel Deaconess Medical Center, Harvard Medical School, Boston, MA USA; 4 Department of Physical Medicine & Rehabilitation, Spaulding Rehabilitation Hospital, Massachusetts General Hospital, Brigham and Women’s Hospital, Harvard Medical School, Boston, MA USA

**Keywords:** Spinal cord injury, Multimodality intervention, Exercise, Androgen therapy

## Abstract

**Background:**

A spinal cord injury (SCI) is a devastating, life-changing event that has profoundly deleterious effects on an individual’s health and well-being. Dysregulation of neuromuscular, cardiometabolic, and endocrine organ systems following an SCI contribute to excess morbidity, mortality and a poor quality of life. As no effective treatments currently exist for SCI, the development of novel strategies to improve the functional and health status of individuals living with SCI are much needed. To address this knowledge gap, the current study will determine whether a Home-Based Multimodality Functional Recovery and Metabolic Health Enhancement Program that consists of functional electrical stimulation of the lower extremity during leg cycling (FES-LC) plus arm ergometry (AE) administered using behavioral motivational strategies, and testosterone therapy, is more efficacious than FES-LC plus AE and placebo in improving aerobic capacity, musculoskeletal health, function, metabolism, and wellbeing in SCI.

**Methods:**

This single-site, randomized, placebo-controlled, parallel group trial will enroll 88 community-dwelling men and women, 19 to 70 years of age, with cervical and thoracic level of SCI, ASIA Impairment Scale grade: A, B, C, or D, 6 months or later after an SCI. Participants randomized to the multimodality intervention will undergo 16 weeks of home-based FES-LC and AE training plus testosterone undecanoate. Testosterone undecanoate injections will be administered by study staff in clinic or by a visiting nurse in the participant’s home. The control group will receive 16 weeks of home-based FES-LC and AE exercise plus placebo injections. The primary outcome of this trial is peak aerobic capacity, measured during an incremental exercise testing protocol. Secondary outcomes include whole body and regional lean and adipose tissue mass; muscle strength and power; insulin sensitivity, lipids, and inflammatory markers; SCI functional index and wellbeing (mood, anxiety, pain, life satisfaction and depressive symptoms); and safety.

**Discussion:**

We anticipate that a multimodality intervention that simultaneously addresses multiple physiological impairments in SCI will result in increased aerobic capacity and greater improvements in other musculoskeletal, metabolic, functional and patient-reported outcomes compared to the control intervention. The findings of this study will have important implications for improving the care of people living with an SCI.

**Trial registration:**

ClinicalTrials.gov:  (NCT03576001).

Prospectively registered: July 3, 2018.

## Background

A spinal cord injury (SCI) is a devastating, life-changing event that results in paralysis, reduced physical activity and accelerated morbidity and mortality [1, 2]. The inter-linked dysregulation of cardiometabolic, musculoskeletal and endocrine bodily organs that rapidly occurs following immobilization after SCI contributes to impaired cardiorespiratory function, osteoporosis, fractures, changes in body composition, insulin resistance, deficits of anabolic hormones, autonomic dysfunction and chronic inflammation [2–11]. Depressive symptoms, anxiety, and chronic pain further contribute to poor quality of life among individuals living with an SCI [12–14].

Despite its profoundly deleterious effects on an individual’s health and well-being, no effective treatments currently exist for SCI. Established exercise interventions for SCI, such as functional electrical stimulation during leg cycling (FES-LC) and arm ergometry exercise (AE) with or without stimulation, are feasible, but only modestly efficacious when administered alone and have had limited impact on overall function and health outcomes [15–18]. This is largely because the training workrates achieved by FES-LC and AE alone in SCI individuals are typically of insufficient intensity to induce meaningful physiological adaptations. Systematic reviews have also highlighted the low quality of evidence due to the well-recognized challenges in conducting exercise studies in people with SCI; not surprisingly, many intervention trials have not been randomized, or have not included a matched control group, or have been limited by a small sample size [16, 19–21]. Furthermore, most exercise interventions for SCI are typically conducted in specialized rehabilitation centers, posing significant accessibility and adherence challenges for a patient population that already faces many physical, psychosocial and environmental barriers to participating in exercise programs [22, 23].

The National Center for Medical Rehabilitation Research of the National Institutes of Health has deemed the development of multimodality interventions to comprehensively improve the functional and health outcomes, and quality of life of individuals living with SCI a high priority. The current study addresses this knowledge gap and attempts to overcome the limitations of many prior exercise studies in SCI. This randomized placebo-controlled trial will test the hypothesis that an innovative Home-Based Multimodality Functional Recovery and Metabolic Health Enhancement Program, that simultaneously incorporates FES-LC plus AE administered using a behavioral motivational strategy, and testosterone treatment, is more efficacious than a control intervention of FES-LC, AE and placebo. We hypothesize that FES-LC plus AE leads to higher levels of exercise intensity [15, 24, 25], and the induced training effects will be augmented by testosterone administration in people with SCI [26–29]. The primary outcome of this study is the change in peak aerobic capacity. Testosterone supplementation has been shown to increase hemoglobin, tissue capillarity, mitochondrial biogenesis and mitochondrial quality, all of which would be expected to improve tissue oxygen delivery and aerobic capacity [30–33]. We have shown previously that the anabolic effects of testosterone on muscle are augmented by exercise training^,^ which may further enhance the improvements in physical capacity in participants randomized to the multimodality intervention [28, 34]. In addition, because androgen deficiency is highly prevalent in SCI and contributes to the loss of muscle mass and strength, metabolic dysregulation, fatigue and low mood [35–37], we will also determine whether the multimodality intervention that includes exercise training and testosterone treatment induces greater gains in measures of musculoskeletal health, metabolism, function and well-being in SCI.

## Methods

### Study design

**Fig. 1:**
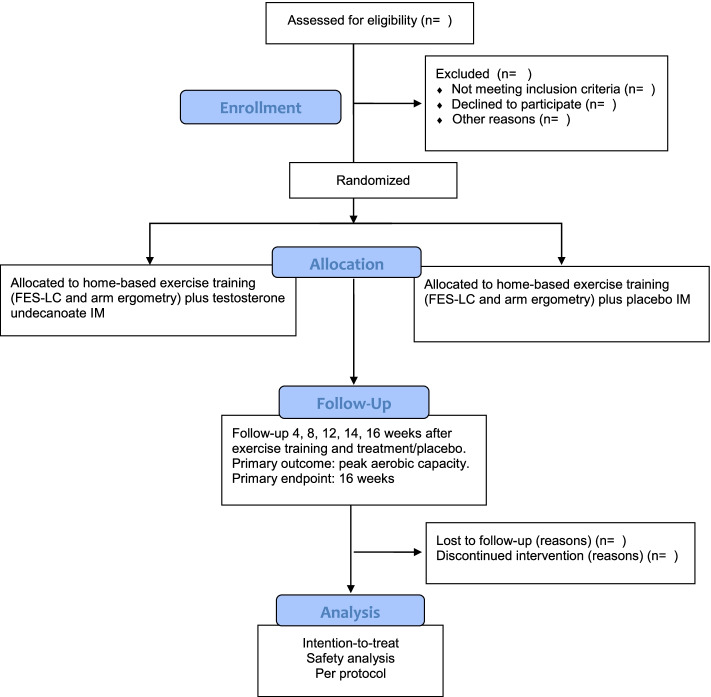
Flowchart of trial participation

This phase 2, proof-of-concept study is a randomized, placebo-controlled, double-blind parallel group trial in community dwelling adults with SCI (Fig. 1). The protocol adheres to the SPIRIT guidelines and the trial reporting will be guided by the Consolidated Standards of Reporting Trials (CONSORT) Statement (Fig. 1) [38, 39]. The study was approved by the Institutional Review Board for Human Subjects Research of the Mass General Brigham healthcare System and registered at ClinicalTrials.gov (NCT03576001).

### Study setting

This study is being conducted at a single academic medical center: Brigham and Women’s Hospital, Boston, MA, USA. Participants who meet eligibility criteria will also be recruited from the SCI Center at Spaulding Rehabilitation Hospital, Boston, MA.

### Study participants

The study’s inclusion and exclusion criteria are detailed in Table 1. Briefly, we will enroll 88 community dwelling men and women with SCI, age 19 to 70 years, with motor C7-T12 cervical and thoracic lesions, and American Spinal Injury Association Impairment Scale (AIS) grade A, B, C, or D, 6 months or later after an SCI. The participants must be able to provide informed consent, ambulate by wheelchair, perform arm ergometry and FES-LC, and participate in assessments of aerobic capacity and muscle performance. The participants with musculoskeletal conditions (such as advanced rotator cuff pathology or carpal tunnel syndrome) or neurological disorder that would prevent them from performing the prescribed arm ergometry; current fractures in the upper and lower extremity; a contraindication for testosterone treatment in accordance with the Endocrine Society guidelines [40]; conditions that would render exercise unsafe or unfeasible such a severe autonomic dysreflexia, severe pressure sores, severe spasticity and severe pain; body mass index (BMI) > 45 kg/m^2^; use of testosterone or other anabolic therapies, including DHEA and androstenedione, or rhGH in the preceding 6 months; psychosis, bipolar disorder, or major untreated depression; dementia (Mini-Mental State Examination score < 24); or myocardial infarction or stroke within 3 months of entry will be excluded. Also excluded are persons with eGFR of < 50 ml/min/ 1.73 m2; ALT and AST > 3 x upper limit of normal; hemoglobin (Hb)-A1c > 9.0% or diabetes requiring insulin therapy. The participants who enroll in the study will be advised not to participate in any other exercise program other than that prescribed in the study protocol and not to make major changes to their dietary intake throughout the course of the study.

**Table 1:** Study inclusion and exclusion criteria

**Inclusion Criteria**	
1. Men and women, 19 to 70 years	
2. Confirmed cervical and thoracic, AIS A-D who are at least 6 months post-injury	
3. Uses a wheelchair as their primary mobility mode	
4. Medically stable, able to follow directions	
5. Able to provide informed consent	
6. For females of reproductive potential who are sexually active: use of highly effective contraception for at least 1 month prior to Day 1 and agreement to use such a method during study participation and for an additional 12 weeks after the end of intervention	
**Exclusion Criteria**	
1. Upper extremity musculoskeletal conditions (such as advanced rotator cuff pathology or carpal tunnel syndrome) or neurological disorder that would prevent the participant from performing the prescribed arm ergometry	
2. Current fractures in the upper and lower extremity	
3. Individuals with a contraindication for androgen (in accordance with the Endocrine Society and ISSAM Guidelines)	
4. Conditions that would render exercise and FES unsafe or unfeasible such as severe autonomic dysreflexia, severe pressure sores, severe spasticity and severe pain	
5. Body mass index (BMI) > 45 kg/m^2^	
6. Renal dysfunction as indicated by GFR of < 50 ml/min	
7. Use of testosterone or other anabolic therapies, including DHEA and androstenedione, or rhGH in the preceding 6 months	
8. Active cancer requiring therapy and which may limit life expectancy to less than 5 years	
9. Psychosis, bipolar disorder, or major untreated depression	
10. Dementia (Mini-Mental Status Exam [MMSE] < 24)	
11. Myocardial infarction (MI) or stroke within 3 months of entry	
12. Pacemaker	
13. ALT and AST > 3 x upper limit of normal	
14. Poorly controlled diabetes as indicated by hemoglobin (Hb)-A1c greater than 9.0% or diabetes requiring insulin therapy	
15. Blood thinners such as Coumadin, heparin, rivaroxaban (Xarelto), dabigatran (Pradaxa), lovenox (subcutaneous heparin), apixaban (Eliquis) (aspirin, plavix and other anti-platelet agents are allowed)	
16. Systolic blood pressure (BP) > 170 or diastolic BP > 100 mmHg	
17. Current grade 2 or greater pressure ulcers at relevant contact sites	
18. Pressure sores or open wounds on the areas that restricts their participation	
19. Because the safety of testosterone has not been established in pregnancy and lactation, we will exclude pregnant or lactating women and women of childbearing potential who are sexually active but are unwilling or unable to use a reliable form of contraception.	
20. Participation in a structured exercise program currently or in the past 2 months	

### Randomized allocation of study participants

Study participants will be randomly assigned to the multimodal intervention or the control intervention using a computer-generated concealed block-randomization scheme with randomly varying blocks and stratification for sex (male, female), and age (19 to 44, 45 to 70 years), and AIS score (A, B, C or D). Participants will be assigned to their respective intervention according to the randomization schedule developed by Data Management Center. Participants will be assigned a randomization number in the order in which they are enrolled.

### Masking

The study participants and the study staff will be blinded and unaware of the intervention assignment. Only the dispensing pharmacist and the study biostatistician will have access to the intervention assignment. The syringes containing the testosterone undecanoate and the placebo injections will be masked to maintain blinding.

### Study interventions

#### FES-LC and AE exercise

Functional electrical stimulation leg cycling (FES-LC) and arm cycle ergometry (AE) without stimulation will be performed in the subjects’ homes using the RT300 leg and arm system (Restorative Therapies, Inc. (RTI), Baltimore, MD). Functional electrical stimulation is an established rehabilitation modality used to stimulate lower motor neurons in individuals who may have lost all or some voluntary control of nerves connecting the spinal cord to peripheral skeletal muscle. Electrical stimuli will be conducted to the quadriceps, hamstrings, and gluteii of both legs via surface electrodes. Visual feedback on leg cycling stimulation level, speed, distance and work rate are presented to the user on a display and transmitted to a secure server in real time via the RTI Link data acquisition system. Study staff may access these data either in real time or at any time after a training session. The FES-LC and AE exercise device may be used with the participants’ wheelchair which is securely attached to the bottom of the RT300 frame.

After baseline testing, all subjects will complete their first 1-2 weeks of training sessions in the exercise physiology laboratory under the direct supervision of a staff exercise physiologist (Table 2). Training with be tailored specifically to each individual based on baseline cardiopulmonary exercise tests (CPXT) performed on the RT300 with stimulation parameters adjusted and programmed for each individual. Stimulation parameters for FES-LC include frequency, pulse width, and amplitude. These will be adjusted as necessary during this first week of training to achieve optimal stimulation. Subjects and their caregivers, if available, will be given detailed instructions on electrode application and removal, skin care, and proper use of the FES-LC and AE device used for training. During the initial weeks of training, subjects will attempt to exercise continuously for 20 min at 50% peak work rate achieved in CPXT for aerobic capacity. If 20 minutes of continuous exercise cannot be performed, the subject will be instructed to cycle as long as possible followed by a 4-min rest period until 20 total exercise minutes are accumulated. Rest periods will decrease as tolerated to allow more continuous exercise as subjects accommodate to the training. Supervised laboratory training will continue as needed until participants become independent in use of the exercise device. After the subject has completed this familiarization training phase in the laboratory, the exercise device will be professionally installed in the subjects’ homes. These machines will be pre-programmed with the FES-LC stimulation parameters used at the conclusion of each subject’s laboratory training and the target work rate and duration for the training sessions. Similarly, the arm ergometer will be individually programmed for the target work rate and training duration. An exercise physiologist will visit each subject in his or her home to assist in carrying out the first day of the home training program. Subsequently, remote audio or video monitoring of training will be conducted as necessary.

**Table 2:** 16-week progressive exercise training using FES-LC and AE

	Weeks 1-2	Weeks 3-6	Weeks 7-10	Weeks 11-16
**Location**	Exercise Physiology Laboratory and/or Home	Home		
**Duration FES-LC**	10-20 minutes (in intervals as needed)	20-25 minutes	25-30 minutes	30-40 min as tolerated
**Duration Arm Ergometry**	20 minutes (in intervals as needed)	20-25 minutes	25-30 minutes	30-40 min as tolerated
**Frequency FES-LC and AE**	1-3 days/wk	3 days/wk	3 days/wk	4 days/wk. as tolerated
**Intensity FES-LC and AE**	40-50% WRpeak (RPE: 11-13)	50-60% WRpeak (RPE:12-14)	60-70% WRpeak (RPE: 13-15)	70-80% WRpeak (RPE:13-16)

*FES-LC* functional electrically stimulated leg cycling, *AE* arm ergometry cycle exercise, *WRpeak* peak work rate, *RPE* rate of perceived exertion. Frequency, duration, and intensity will be adjusted by the exercise physiologist according to exercise tolerance

All home exercise training sessions for FES-LC will start with a 3-min motor-assisted warm-up at 35-40 rpm (RPM) with no electrical stimulation; warm up for arm cycling will be performed for 3-min at 50 RPM with no resistance. Since subjects will have varying time since injury (months to several years) and different lesion levels, we will apply the stimulation parameters with a range chosen to accommodate not only entry characteristics but also changes in responsiveness to the stimulation over the 16-week training periods [41]. We will apply frequencies of 10 Hz to a maximum of 100 Hz. Higher frequencies increase force production but also increases fatigue which can limit training duration. Low frequencies are more tolerable in more recently injured individuals as well in those who are initially unresponsive. Pulse width will range between 50 and 500 microseconds. Longer pulse widths evoke greater torque production and can result in tetanic contractions facilitating joint movement. Longer pulse width used with lower frequencies can lead to maximizing torque while minimizing sensation. Amplitude will be set in a range of 0-140 mA per channel adjusted automatically along with flywheel resistance to maintain the target peak work rates. Greater amplitude activates more muscle and increases torque production. However, some spinal cord injured people cannot tolerate pain associated with higher amplitudes. If the target duration cannot be performed continuously, we will allow shorter intervals interspersed with rest to accumulate the exercise duration target with progression to the target duration as tolerated. After warm-up, the resistance will then be increased to the target work rate for both FES-LC and arm ergometry and sustained continuously as tolerated for the target durations in each of the four training phases (Table 2). As with FES-LC, individuals who are unable to maintain continuous arm exercise will be allowed to finish the target duration in interval fashion. Subjects will be instructed to inform the study staff or an exercise physiologist about difficulties in performing continuous exercise for the target duration. In addition, daily review of training data gathered via RTI-Link (pedal frequency, power output (watts), exercise time, motor support, and % stimulation for FES-LC) and training RPE will alert the exercise physiologists to this difficulty followed by appropriate revisions of training routine. All participants will be instructed to complete a 5- to 10-minute program of gentle stretching or flexibility exercises prior to each training session.

#### Training progression

Progression in duration, intensity, and frequency of FES-LC and arm ergometer exercise is described in Table 2. Over the course of the study, duration will increase from 20 minutes to 40 minutes, intensity from 50 to 80% of baseline peak work rate, and weekly training frequency will increase from 3 days/week to 4 days/week. The Borg scale of self-perceived exertion, which ranges from 6 to 20, will also as used to measure intensity of activity and ensure optimal training progression throughout the intervention [42]. As noted in Table 2, the actual targets for duration, intensity and frequency may be adjusted by an exercise physiologist based on subject tolerance to the training parameters.

#### Testosterone / placebo dosing and administration

Testosterone undecanoate injections will be administered by the study personnel and subjects will be observed for 30-minutes. The testosterone undecanoate dose for men (first dose of 750-mg on the day of randomization, second 750-mg dose during week 4, and the third 750-mg dose during week 14) is based on the approved regimen and Endocrine Society guidelines [40, 43]. The dose used in women will be one fourth that used in men (first 180 mg dose on the day of randomization, second 180-mg dose during week 4, and the third 180-mg dose during week 14). A large body of published data have shown the safety and efficacy of testosterone in increasing lean mass and strength, and bone density [44, 45]. The participants randomized to the control group will receive an equal volume of placebo at randomization, week 4 and week 14.

#### Behavioral motivational strategies

Study staff will be trained to use motivational interviewing techniques to enhance overall adherence to the exercise intervention. Prior to participants starting the intervention program, staff will identify relevant social contextual factors (e.g.*,* financial resources) that impact behavior, seek to build a partnership with the participant, and evoke the participant’s own internal motivations for change. Throughout the intervention, study staff will communicate with the participant to set behavior change goals tailored to participant’s ability level. Study staff will discuss obstacles to participants’ long-term participation, have the participant identify strategies to overcome barriers, and refine the exercise goals as necessary. Participants will also be encouraged to call the study staff between sessions if they need assistance. Motivational interviewing-based interventions have been previously implemented among individuals with SCI and have had a positive impact on physical activity levels [46–48].

### Study outcomes

The study outcomes are listed in Table 3.

**Table 3:**
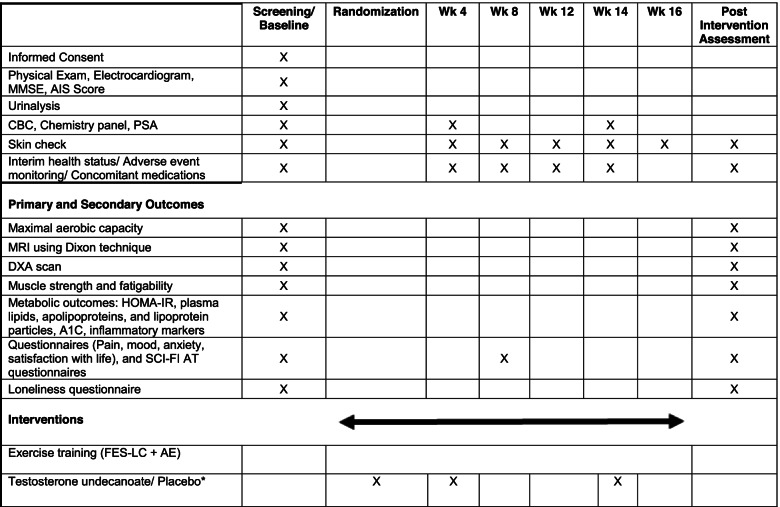
Study assessment and intervention schedule

*MMSE* Mini-mental State Examination, *AIS* American Spinal Injury Association Impairment Scale, *CBC* complete blood count, *PSA* prostate specific antigen, *MRI* magnetic resonance imaging, *DXA* dual-energy X-ray absorptiometry, *SCI-FI AT* adaptive technology version of Spinal cord injury functional index, *FES-LC* functional electrical stimulation leg cycling, *AE* arm ergometry. * Intramuscular injections will be administered at randomization, week 4 and week 14

### Primary outcome

#### Peak aerobic capacity

Cardiopulmonary exercise testing (CPXT) will be used to ascertain peak aerobic capacity separately for FES leg cycling (FES-LC), arm ergometry (AE), and concurrent FES-LC plus AE. Standardized procedures for CPXT will be used for all tests. Subjects will come to the exercise laboratory for equipment and procedure familiarization on a separate day prior to the three tests described above. The FES-LC CPXT will be performed on the RT300 LC system (Restorative Therapies, Inc. (RTI)), Baltimore, MD. Warm-up will consist of 3-minutes of motor-assisted FES-LC with no resistance and no electrical stimulation at a pedal frequency of 30-35 RPM. FES-LC will then be performed with sequential electrical stimulation, via surface electrodes, applied to quadriceps, hamstrings, and gluteii muscles. Stimulation parameters (frequency (Hz); pulse width (ms), amplitude (mA)) will be individualized for each participant during familiarization testing and adjusted to maintain a pedaling frequency of 35-40 RPM without motor support. Work rate will start at 0 watts then increase 0-3 watts per min until attainment of peak exercise capacity, defined as a precipitous decline in power output during maximal leg stimulation. The arm ergometer CPXT will be performed using a Monark 891E arm ergometer (Monark Exercise AB, Vansbro, Sweden) administered 30 minutes after completion of the FES-LC CPXT. Work rates will be incremented by 10-20 watts per min to volitional fatigue. Arm cranking frequency will be set at 50 RPM. No electrical stimulation will be applied during arm ergometer CPXT. On a separate testing day, approximately 2 days but no more than 7 days after the initial FES-LC and AE CPXTs, subjects will perform a hybrid CPXT. The subjects will begin the CPXT test using the arm ergometer. FES-LC will begin after arm exercise has started so that both FES-LC and arm exercise end together. The start time for FES-LC will be determined from the previously completed FES-LC CPXT. A fully automated metabolic measurement system (Medical Graphics Cardio2 Ultima, St. Paul, MN) integrated with an electrocardiograph will be used to assess aerobic capacity and other parameters of aerobic performance.

### Secondary outcomes

#### Body composition

Whole body and regional muscle and fat mass will be assessed by magnetic resonance imaging using a 2-point Dixon technique with a 3D gradient echo sequence on a 3 T scanner (GE Premier), enabling acquisition of high-resolution 3D volumes. Both in-phase, and out of -phase, fat and water images can be generated from a single acquisition. Full body acquisition will be facilitated using a 20 channel adaptive image receive (AIR) coil to image the torso and progressively moving the coil to cover the lower extremities. Images will be acquired using two different three-dimensional gradient echo 2 point and 3 point Dixon-based sequences: LAVA Flex (echo time/repetition time (TE/TR) = 2.0/4.9 ms, flip angle = 12°, receiver bandwidth = 142.86 kHz, slice thickness = 8 mm, matrix size = 300 × 224, acquisition time = 18 s) and IDEAL IQ (TE/TR = 3.2/8.4 ms, flip angle = 4°, receiver bandwidth = 142.86 kHz, slice thickness = 8 mm, matrix size = 192 × 188, acquisition time = 41 s, respectively. Both sequences provide fat only and water only images. Different fat compartments will be quantified using non-linear segmentation, based on the Dixon method. Body composition and fat mass will also be measured by dual-energy X-ray absorptiometry (DXA).

#### Upper body strength and fatigability

Muscle strength in the upper body will be measured with the seated chest press exercise using a pneumatic resistance (Keiser Corporation, Fresno, CA) and the 1-repetition maximum method (1-RM) [29, 49]. Subjects will transfer to the seat on the chest press machine with assistance. A shoulder harness and seat belt will be applied to help stabilize the subject and the feet will be supported. Seat height, handle position, and full range of motion will be standardized. Subjects will be familiarized with the exercise, practice the technique, and complete a brief low resistance warm-up. The 1-RM procedure consists of a warmup set with 5 to 8 repetitions at a resistance set to about 50% of the participant’s estimated 1-RM. The test progresses with increasing loads interspersed with standardized rest periods until the subject can perform only one full range-of-motion repetition in good form. Load increments are determined by subject perception of effort and the examiner’s experience. The participants will be tested twice on nonconsecutive days with the better of the 2 trials reported as the 1-RM. Fatigability in the chest press exercise will be assessed on the same machine and subject positioning used for the 1-RM tests. Subjects will perform as many full range of motion, continuous repetitions as possible using good form. The load for this test will be 80% of the individual’s 1-RM [50].

#### Circulating metabolic profile

Fasting blood samples will be obtained to determine measures that will include glucose, hemoglobin A1C, insulin sensitivity using the homeostasis model assessment-estimated insulin resistance (HOMA-IR) [51], plasma lipids, apolipoproteins A, B and CIII, and lipoprotein particles as markers of atherogenicity, and inflammatory markers hsCRP and IL-6.

#### Patient-reported outcomes to assess function and wellbeing

The Spinal Cord Injury-Functional Index/Assistive Technology (SCI-FI/AT) will be used to measure mobility, fine motor function, ambulation, and wheelchair mobility [52, 53]. Wellbeing will be assessed using validated measures of pain, anxiety depressive symptoms, loneliness, and life satisfaction. The Modified Brief Pain Inventory (BPI) will be used to assess pain intensity (sensory dimension) and interference with function (reactive dimension) [54, 55]. The Patient Health Questionnaire (PHQ-9), a 9-item scale will be used to assess mood and depressive symptoms [56]. We will assess anxiety using the generalized anxiety disorder (GAD-7) questionnaire [57]. Loneliness will be assessed using the Three-Item Loneliness Scale and the Satisfaction with Life Scale will be utilized to assess happiness with life [58, 59].

#### Safety assessment and monitoring

We will incorporate a standardized safety monitoring plan, as recommended by the Endocrine Society guidelines [40]. Safety assessment will include a listing of all adverse and serious adverse events; injuries, including those incurred during exercise training; number of persons with erythrocytosis; number of persons referred for prostate biopsy; prostate-related adverse events; and major adverse cardiovascular events. Safety laboratory tests including blood counts and chemistries will be evaluated at baseline and throughout the intervention period. Events will be classified according to the Medical Dictionary for Regulatory Activities (MedDRA) system organ class coding system [60]. An external data safety monitoring board (DSMB) will monitor the study in accordance with National Institute of Child Health and Human Development (NICHD) / National Institutes of Health (NIH) guidelines. The DSMB consists of individuals with expertise in SCI, clinical trials and biostatistical analysis of clinical trials data.

#### Sample size calculation

A sample size of 88 subjects was based on the following considerations: a type-I error probability 0.05; randomization in a 1:1 ratio and stratification by sex, age (19-44, 45-70 yrs), and AIS level (A, B, C, or D); a loss-to-follow-up rate of up to 20%. There are no published trials of the combined administration of an androgen and FES-LC plus AE in patients with SCI. Therefore, the sample size estimates were guided by data on the effects of exercise in patients with SCI, the effects of testosterone in hypogonadal men and in patients with SCI, and the effects of testosterone undecanoate in other populations. The gains in aerobic capacity have varied substantially across trials but have averaged around 10 to 14% [16, 19–21]. Based on previous studies, we hypothesize that the mean gain attributable to exercise will be ~ 12%; we anticipate that addition of testosterone undecanoate exercise will augment the treatment effect and that the mean gain in peak oxygen uptake attributable to the multimodality intervention will be 20%. Thus, conservatively, the treatment effect will be 8% improvement in peak aerobic capacity (SD no greater than 11%). Under the assumed effects described above, simulation studies estimated that the proposed primary mixed-effects analysis for continuous endpoint will have ~ 90% power to detect an effect size f = 0.75 SD with conservative assumption of within subject autocorrelation, rho = 0.45.These changes are clinically meaningful because gains in aerobic capacity of 6-10% have been associated with improvements in outcomes in patients with SCI and COPD [16, 19–21, 28].

For all secondary outcomes, the trial sample size of 88 participants has sufficient power to detect similar effect sizes or larger between the multimodality intervention vs. control intervention i.e.: to detect the hypothesized difference of 2.0 kg (SD 2.6 kg) between arms in change from baseline for lean body mass. Furthermore, we anticipate that multimodality intervention will improve insulin sensitivity by ~ 30%, and the exercise alone by ~ 10%, hence a subsample of 30 completers has at least 80% power to detect this difference of 20% between intervention arms (SD no more than 18%). These assumptions are reasonable because in previous studies, FES and cycle ergometry have been shown to improve HOMA by ~ 25% [15].

#### Statistical analyses

The analysis of covariance and linear mixed-effect regression models, controlling for stratification factors, will be employed to formally estimate intervention effects for primary and secondary outcomes. Mixed model regressions will include factors for treatment, visit and visit-by-treatment interaction. The unstructured covariance matrix will be assumed, however if models do not converge, compound symmetry will be employed. Model-based point estimates of change from baseline in endpoints will be accompanied by 95% confidence intervals. Participants will be analyzed according to intent-to-treat principle. A safety analysis set will consist of all subjects who are enrolled and received the investigational product (testosterone undecanoate or placebo), including subjects who do not complete all scheduled study visits. In addition, a per-protocol analysis will be considered on subset of the participants who were compliant and finished the study. For primary and pre-specified secondary hypotheses, no type 1 error adjustment is planned, and all *p*-values will be considered nominal.

#### Current enrollment status and COVID-19 modifications

The trial began recruitment in June 2019. To date, 41 participants have attended an in-person screening visit, of whom 25 have been randomized to the multimodality or control intervention. Due to the COVID-19 pandemic, study recruitment was suspended in March 2020 and 3 participants discontinued their participation in the study. Study recruitment and trial activities restarted in August 2020 and several trial modifications were made to maintain enrolment, the safety of study participants and staff, compliance with institutional policies and good clinical research practice. The timeline of study assessments was changed to decrease the number of times a participant was required to attend the clinic (Week 8 and week 12 in-person assessments were changed to virtual visits). Specific risk mitigation strategies were also employed to maximize participant and staff safety during study assessments and participant familiarization sessions. These strategies included the use of standardized pre-screening for COVID symptoms, personal hygiene and the use of personal protective equipment, optimization of ventilation in the exercise physiology laboratory and reconfiguration of equipment to allow for adequate physical distancing [61]. Study recruitment is estimated to be completed in January 2024 with study interventions and data collection finalized by May 2024.

#### Dissemination

This study will comply with the National Institutes of Health (NIH) Public Access Policy, Data Sharing Policy, Policy on the Dissemination of NIH-Funded Clinical Trial Information and the Clinical Trials Registration and Results Information Submission rule. Regardless of statistical significance, trial results will be presented at national and international conferences and published in peer-reviewed journals. Upon acceptance for publication, these manuscripts will also be submitted to the digital archive PubMed Central. Following publication of the primary manuscript, participants will be informed of their group allocation and provided with the study results. This trial is registered at ClinicalTrials.gov and results will also be submitted to ClinicalTrials.gov.

## Discussion

**Fig. 2:**
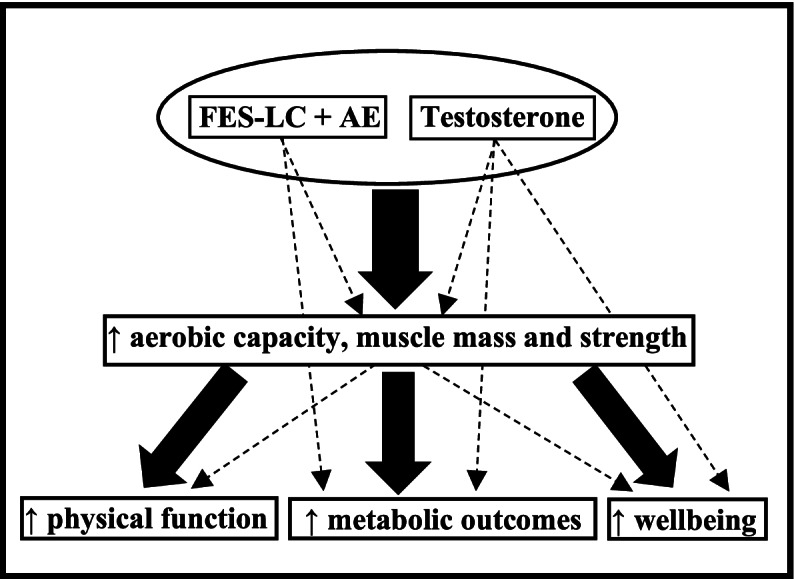
Conceptual Model for Hypothesized Effects of Multimodality Intervention in SCI. Exercise (FES-LC + AE) and testosterone may individually increase aerobic capacity, and muscle mass and strength, which would improve physical function and wellbeing. Exercise or testosterone may also enhance metabolic adaptations *directly* through effects on muscle mass and contractility mediated via myokines, fuel utilization, and other mechanisms, and indirectly via increased activity. Testosterone may directly affect metabolism and wellbeing. When combined, the synergistic effects of the multimodality intervention (FES-LC + AE + testosterone) may induce greater benefits compared to each intervention alone (as shown by larger solid arrows)

The proposed trial (FIT-SCI) is an important proof-of-concept study that will determine the efficacy a novel home-based multimodality function promoting strategy for individuals living with SCI. We anticipate that a multimodality intervention which combines FES-LC, AE and androgen therapy and simultaneously addresses multiple pathophysiologic factors in SCI, will result in greater improvements in aerobic capacity, musculoskeletal health, metabolism and function compared to FES-LC and AE alone (Fig. 2). Important strengths of our study include the deployment of a scalable home-based multimodality intervention, a scientifically rigorous clinical trial design, state-of-the-art assessments, and the inclusion of both performance-based and patient-reported efficacy outcomes. The sample size of 88 participants was rationally guided by consideration of treatment effect and statistical power. The findings from this study will substantially advance our understanding of how to design and implement more effective interventions in SCI. The results of this study will also generate findings that may have important therapeutic and policy implications for improving the care, health and well-being of persons living with SCI.

## Data Availability

Not applicable.

## Abbreviations

AE: Arm ergometry exercise
AIS: American Spinal Injury Association Impairment Scale
BMI: Body mass index
BPI: Modified Brief Pain Inventory
CBC: Complete blood count
COVID-19: Coronavirus disease of 2019
CPXT: Cardiopulmonary exercise test
DXA: Dual-energy X-ray absorptiometry
FES-LC: Functional electrical stimulation during leg cycling
FIT-SCI: A multimodality intervention to improve musculoskeletal health, function, metabolism, and well-being in spinal cord injury
GAD-7: Generalized anxiety disorder questionnaire
HOMA-IR: Homeostasis model assessment-estimated insulin resistance
MedDRA: Medical Dictionary for Regulatory Activities
MMSE: Mini-mental State Examination
MRI: Magnetic resonance imaging
NICHD: Eunice Kennedy Shriver National Institute of Child Health and Human Development
NIH: National Institutes of Health
PSA: Prostate specific antigen
PHQ-9: Patient Health Questionnaire
RPM: Revolutions per minute
RTI: Restorative Therapies, Inc.
1-RM: 1-repetition maximum
SCI: Spinal cord injury
SCI-FI AT: Adaptive technology version of spinal cord injury functional index
